# Chlorogenic Acid Enhances the Intestinal Health of Weaned Piglets by Inhibiting the TLR4/NF-κB Pathway and Activating the Nrf2 Pathway

**DOI:** 10.3390/ijms25189954

**Published:** 2024-09-15

**Authors:** Beibei Zhang, Min Tian, Jing Wu, Yueqin Qiu, Xiaoming Xu, Chaoyang Tian, Jing Hou, Li Wang, Kaiguo Gao, Xuefen Yang, Zongyong Jiang

**Affiliations:** 1 Institute of Animal Science, Guangdong Academy of Agricultural Sciences, Guangzhou 510640, China; 2 State Key Laboratory of Swine and Poultry Breeding Industry, Guangzhou 510640, China; 3 Key Laboratory of Animal Nutrition and Feed Science in South China, Ministry of Agriculture and Rural Affairs, Guangzhou 510640, China; 4 Guangdong Provincial Key Laboratory of Animal Breeding and Nutrition, Guangzhou 510640, China

**Keywords:** weaned piglets, chlorogenic acid, intestinal health, inflammation, oxidative stress

## Abstract

Chlorogenic acid (CGA) is a natural polyphenol with potent antioxidant and anti-inflammatory activities. However, the exact role of it in regulating intestinal health under oxidative stress is not fully understood. This study aims to investigate the effects of dietary CGA supplementation on the intestinal health of weaned piglets under oxidative stress, and to explore its regulatory mechanism. Twenty-four piglets were randomly divided into two groups and fed either a basal diet (CON) or a basal diet supplemented with 200 mg/kg CGA (CGA). CGA reduced the diarrhea rate, increased the villus height in the jejunum, and decreased the crypt depth in the duodenum, jejunum, and ileum of the weaned piglets (*p* < 0.05). Moreover, CGA increased the protein abundance of Claudin-1, Occludin, and zonula occludens (ZO)-1 in the jejunum and ileum (*p* < 0.05). In addition, CGA increased the mRNA expression of pBD2 in the jejunum, and pBD1 and pBD2 in the ileum (*p* < 0.05). The results of 16S rRNA sequencing showed that CGA altered the ileal microbiota composition and increased the relative abundance of *Lactobacillus reuteri* and *Lactobacillus pontis* (*p* < 0.05). Consistently, the findings suggested that the enhancement of the intestinal barrier in piglets was associated with increased concentrations of T-AOC, IL-22, and sIgA in the serum and T-AOC, T-SOD, and sIgA in the jejunum, as well as T-AOC and CAT in the ileum caused by CGA (*p* < 0.05). Meanwhile, CGA decreased the concentrations of MDA, IL-1β, IL-6, and TNF-α in the serum and jejunum and IL-1β and IL-6 in the ileum (*p* < 0.05). Importantly, this study found that CGA alleviated intestinal inflammation and oxidative stress in the piglets by inhibiting the TLR4/NF-κB signaling pathway and activating the Nrf2 signaling pathway. These findings showed that CGA enhances the intestinal health of weaned piglets by inhibiting the TLR4/NF-κB pathway and activating the Nrf2 pathway.

## 1. Introduction

Early weaning has become a universal technique in pig production, which shortens the production cycle of pigs and improves the breeding efficiency. However, early weaning exposes piglets to sudden maternal deprivation, dietary changes, and social stress from mixing multiple litters, resulting in weaning stress [1]. Due to their incomplete development and continuous exposure to various stimuli from the intestinal tract, the intestines of piglets are more susceptible to weaning stress than other tissues [2,3,4]. Weaning stress is highly susceptible to inducing oxidative stress and immune-inflammatory responses in the intestine of piglets, thus impairing their digestion and absorption of nutrients [5,6]. Accumulating evidence shows that weaning stress leads to declined feed intake, diarrhea, growth retardation, and even the death of piglets, which ultimately results in great economic losses in livestock production [7]. Thus, it is crucial to reduce the negative effects of weaning stress on piglets.

A variety of natural antioxidant extracts have been used to protect piglets from weaning stress in intensive production, such as vitamin E, vitamin C, and polyphenols [8,9,10]. Chlorogenic acid (CGA) is one of the most abundant polyphenolic compounds in nature, formed by the esterification of caffeic acid and quinic acid [10,11,12,13]. Previous studies have shown that CGA has a wide range of biological activities, including antioxidant, anti-inflammatory, antibacterial, and glucose and lipid metabolism-regulating functions [10,11]. These characteristics suggest that CGA has broad application prospects in the fields of animal production and even human health care. Recently, CGA has been used as a feed additive to improve the antioxidant capacity in weanling pigs, growing–finishing pigs and sows [14,15,16,17,18,19]. An earlier study showed that a sow diet supplemented with 300 mg/kg CGA during the lactation period improved the plasma antioxidant capacity in sows and increased the birth weights of newborn pigs [20]. In a 14-day trial, Chen et al. (2018a) found that dietary supplementation with 250 mg/kg and 500 mg/kg CGA had no significant effect on piglet growth performance, whereas a diet of 1000 mg/kg CGA significantly reduced the diarrhea incidence and improved the growth performance of piglets [14]. It can be inferred that the effectiveness of CGA may be related to the dosage and duration of its addition in the diet. Further research results indicated that this is related to the improved antioxidant capacity of piglets [14,15]. However, to the best of our knowledge, the potential mechanisms by which CGA improves the intestinal health of weaned pigs have not been fully elucidated. Therefore, the aims of this study were to investigate the effects of CGA on intestinal oxidative stress, the immune-inflammatory response, and intestinal barrier function in weaned piglets and to explore their regulatory mechanisms, with a view to providing nutritional strategies to alleviate weaning stress in piglets.

## 2. Results

### 2.1. Growth Performance

As shown in Table 1, the ADG of the piglets in the CGA group increased by 23.3% compared to those in the CON group, showing a tendency to increase significantly (*p* = 0.096). The diarrhea rate of the piglets in the CGA group was significantly lower than that in the CON group (*p* < 0.05). There was no significant difference in the final weight, ADFI, and G/F between the CON and CGA groups of piglets.

### 2.2. Intestinal Morphology

**Figure 1:**
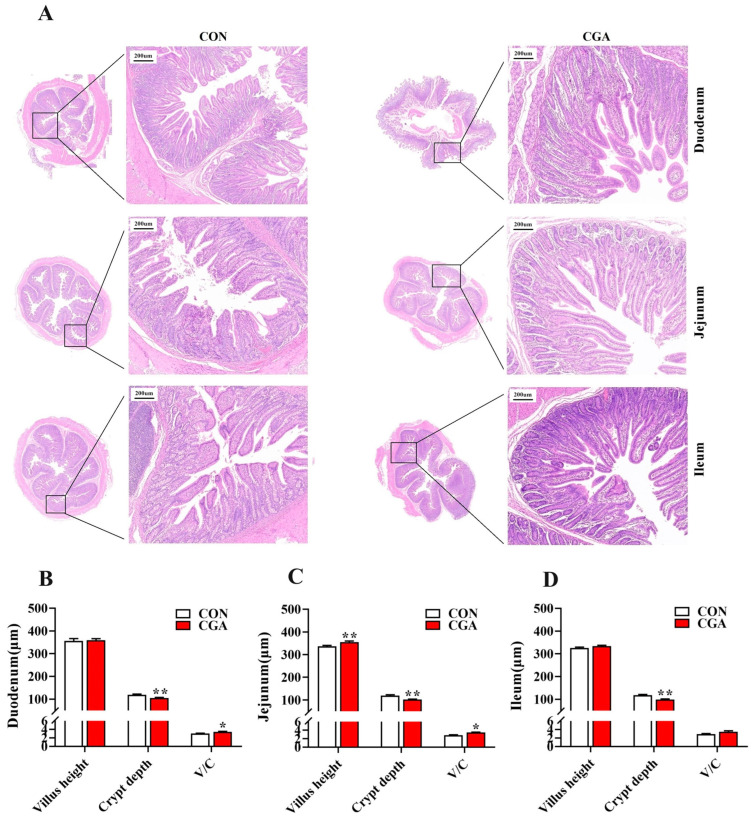
Effects of dietary supplementation with CGA on the villi height and crypt depth of the small intestine in piglets. (**A**) H&E staining of the intestine (scale bar, 500 μm); (**B**–**D**) analysis of villi height and crypt depth in the intestine. Values are presented as the mean ± SEM (*n* = 6). *, 0.01 < *p* ≤ 0.05. **, *p* ≤ 0.01.

The villus height and crypt depth of the duodenum, jejunum, and ileum are shown in Figure 1. In the duodenum (Figure 1B), the crypt depth of the piglets in the CGA group was significantly lower than that in the CON group (*p* < 0.05), and the V/C in the CGA group was significantly higher than that in the CON group (*p* < 0.05). In the jejunum (Figure 1C), the villus height and V/C of the piglets in the CGA group were significantly higher than those in the CON group, and the crypt depth in the CGA group was significantly lower than that in the CON group. In the ileum (Figure 1D), the crypt depth of the piglets in the CGA group was significantly lower than that in the CON group (*p* < 0.05).

### 2.3. Expression of Tight Junctions in the Jejunum and Ileum

**Figure 2:**
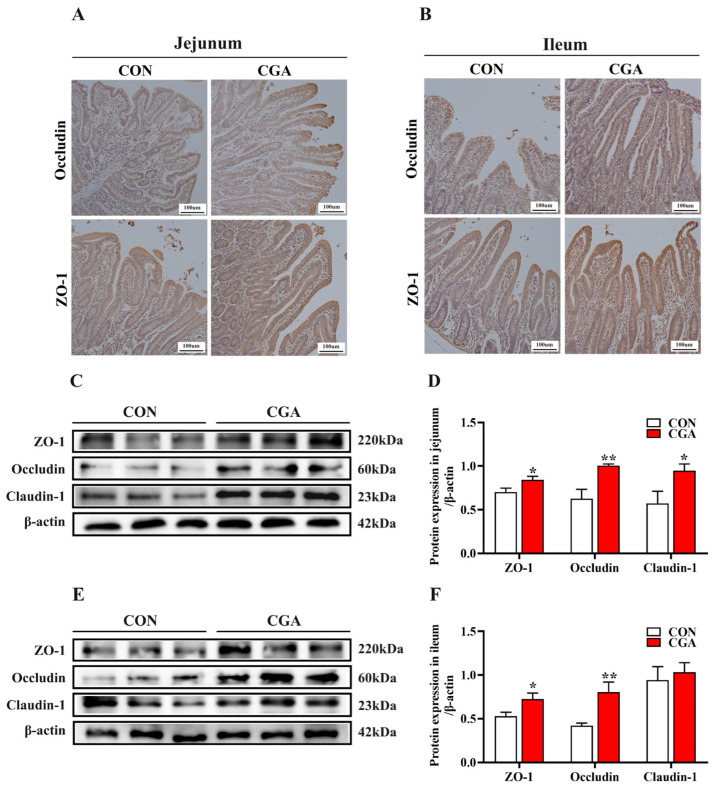
Effects of dietary CGA supplementation on the expression of tight junctions in the jejunum and ileum. (**A**,**B**) The immunoreactivity of tight junctions in the jejunum and ileum of piglets; (**C**–**F**) Western blot analysis of tight junctions in the jejunum and ileum of piglets. *n* = 6. *, 0.01 < *p* ≤ 0.05. **, *p* ≤ 0.01.

Tight junctions serve as crucial indicators for evaluating the intestinal integrity of piglets. The expression of tight junctions in the jejunum and ileum are shown in Figure 2. As shown in Figure 2A,B, the results of the immunohistochemistry analyses indicated that the protein abundance of Occludin and zonula occludens (ZO)-1 in the jejunum and ileum of the piglets in the CGA group was higher than that in the CON group (*p* < 0.05). Consistently, the results of the Western blot analysis showed that the relative protein abundance of Claudin-1, Occludin, and ZO-1 in the jejunum of the piglets in the CGA group was significantly higher than that in the CON group (*p* < 0.05). Moreover, the relative protein abundance of Occludin and ZO-1 in the ileum of the piglets in the CGA group was significantly higher than that in the CON group (*p* < 0.05).

### 2.4. mRNA Expression of Porcine Beta Defensins and Mucins

**Figure 3:**
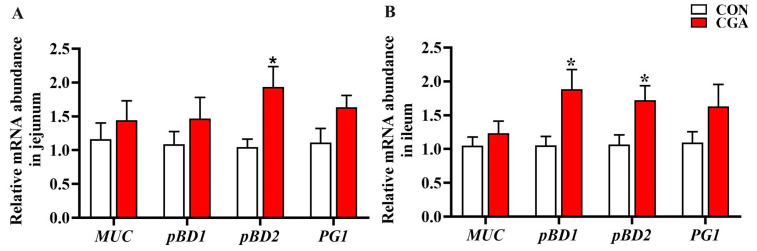
Effects of dietary CGA supplementation on the mRNA expression of mucins and porcine beta defensins in the jejunum (**A**) and ileum (**B**) mucosa of piglets. MUC, mucin; pBD, porcine beta defensins; PG1, Protegrin-1. Values are presented as the mean ± SEM (*n* = 6). *, 0.01 < *p* ≤ 0.05.

The mRNA expression of porcine beta defensins (pBDs) and mucins are shown in Figure 3. In the jejunum (Figure 3A), the pBD2 mRNA expression in the CGA group was significantly higher than that in the CON group (*p* < 0.05). In the ileum (Figure 3B), the mRNA expression of pBD1 and pBD2 in the CGA group was significantly higher than that in the CON group (*p* < 0.05). There was no significant difference in the mRNA expression of MUC between the two groups in the jejunum and ileum (*p* > 0.10).

### 2.5. Microbes in the Ileal Contents of the Piglets

**Figure 4:**
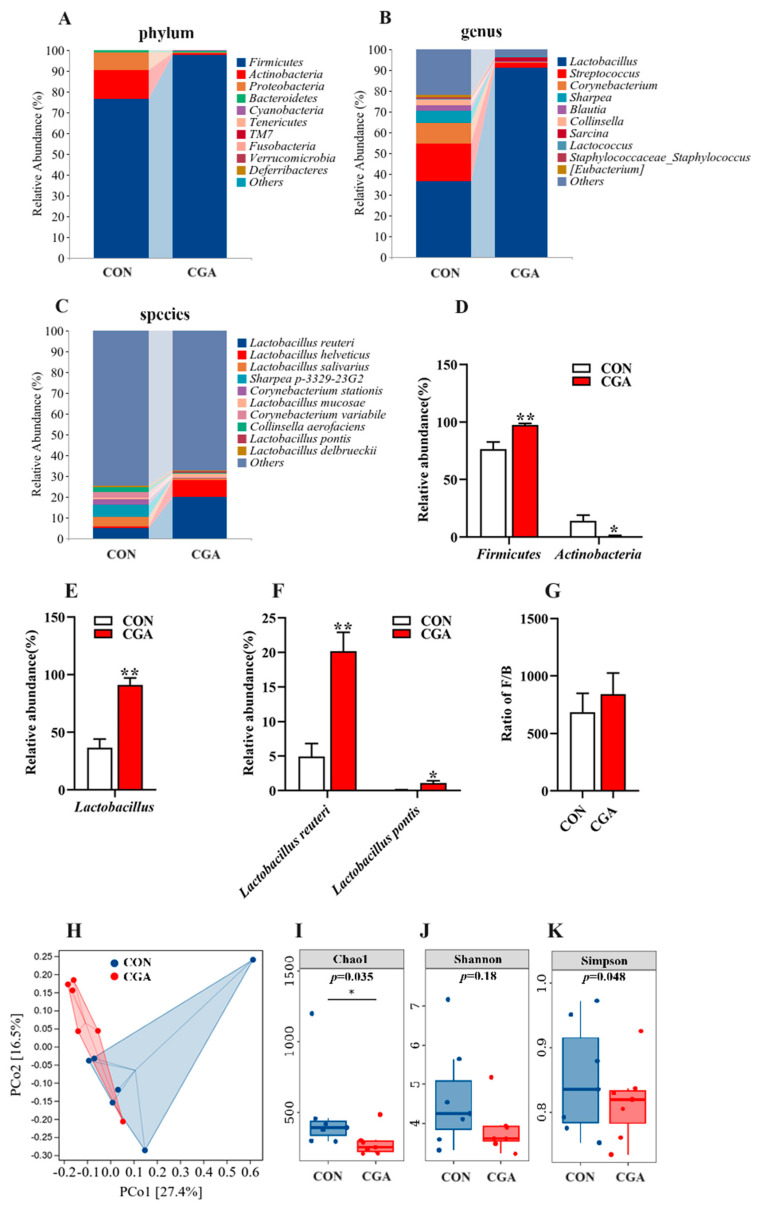
Effects of dietary CGA supplementation on the ileal microbiota of piglets. (**A**–**G**) Relative abundance of microbiota at the phylum, genus, and species levels. (**H**) Beta diversity; (**I**–**K**) alpha diversity. Values are presented as the mean ± SEM (*n* = 6). *, 0.01 < *p* ≤ 0.05. **, *p* ≤ 0.01.

A total of 3,122,363 effective sequences were successfully generated from 12 ileal content samples (two groups, *n* = 6), with an average of 63,722 sequences per sample, and 14,336 OTUs were obtained for further analysis. The sequencing data are accessible online at the National Genomics Data Center (Beijing, China) with the accession number CRA017663. Three major bacterial phyla in the ileal contents of the piglets were Firmicutes (76.38% or 97.60%), Actinobacteria (14.03% or 0.98%), and Proteobacteria (8.30% or 0.19%), accounting for over 98% of the total bacterial community. As shown in Figure 4, the relative abundance of Firmicutes in the CGA group was significantly higher than that in the CON group (*p* < 0.05), while the relative abundance of Actinobacteria in the CGA group was significantly lower than that in the CON group (*p* < 0.05). Within the changes in Firmicutes (Figure 4B,E), the relative abundance of Lactobacillus in the CGA group was significantly higher than that in the CON group (*p* < 0.05). At the genus level (Figure 4C,F), the major sources of changes in Lactobacillus were *Lactobacillus reuteri* and *Lactobacillus pontis*. The relative abundance of *Lactobacillus reuteri* and *Lactobacillus pontis* in the CGA group was significantly higher than that in the CON group (*p* < 0.05). For beta diversity (Figure 4H), the PCoA (Bray–Curtis and Jaccard distance) analysis result showed that the microbiota in the CGA group tended to clearly separate from the CON group. For the alpha diversity (Figure 4I–K), the ileal microbiota were indicated by the Chao1, Simpson, and Shannon indexes. The Chao1 index in the CGA group was significantly lower than that in the CON group (*p* < 0.05), while there were no significant differences in the Simpson and Shannon indexes.

### 2.6. Antioxidant Status and Immune-Inflammatory Profiles in the Serum and Intestine of Piglets

**Figure 5:**
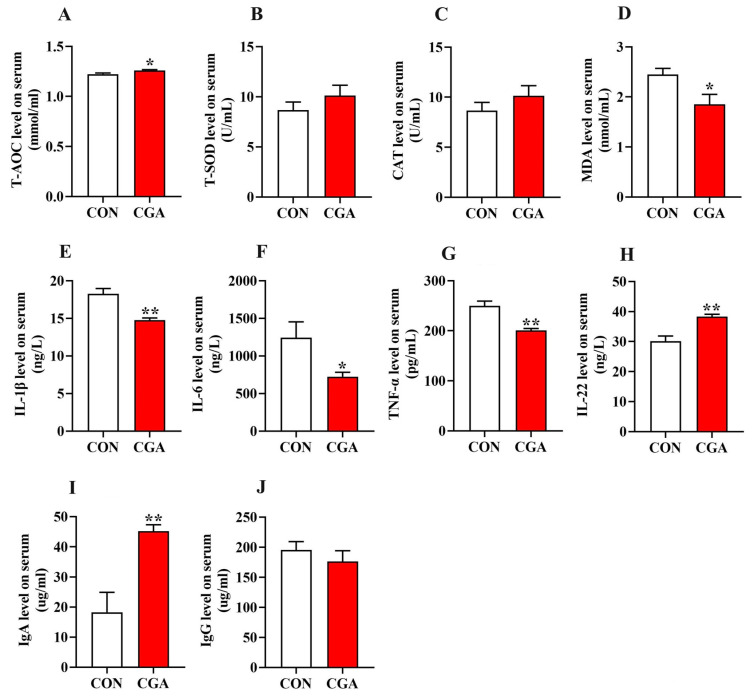
Effects of dietary supplementation with CGA on antioxidant status and immune-inflammatory level in the serum of piglets. (**A**–**D**) The antioxidative and oxidative indicators in the serum. (**E**–**H**); the concentration of inflammatory factors in the serum; (**I**,**J**) the immunoglobulin concentration in the serum. Values are presented as the mean ± SEM (*n* = 6). *, 0.01 < *p* ≤ 0.05. **, *p* ≤ 0.01.

**Figure 6:**
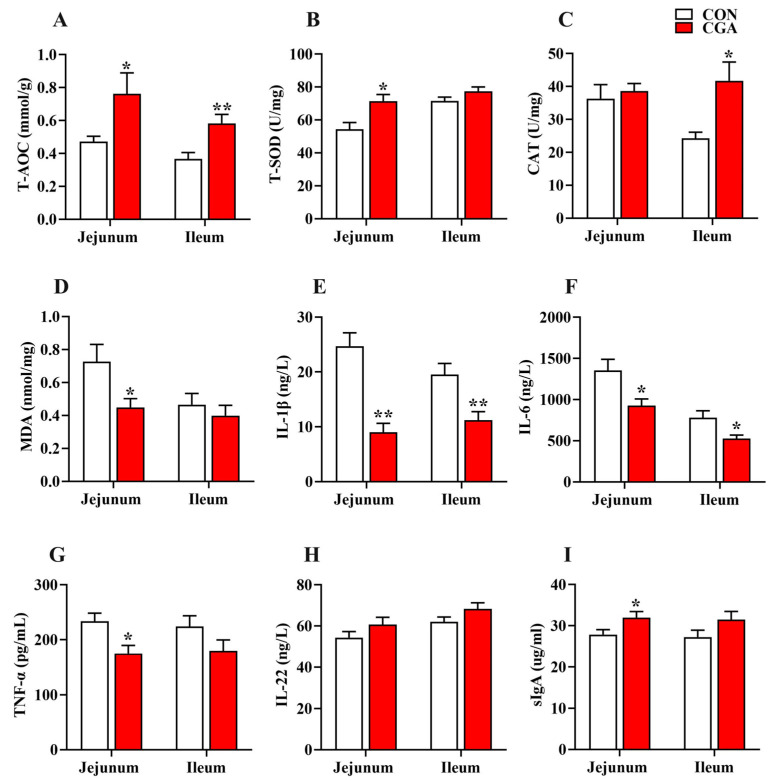
Effects of dietary supplementation with CGA on antioxidant status and immune-inflammatory level in the jejunum and ileum of piglets. (**A**–**D**) The antioxidative and oxidative indicators in the serum; (**E**–**H**) the concentration of inflammatory factors in the serum; (**I**) the immunoglobulin concentration in the serum. Values are presented as the mean ± SEM (*n* = 6). *, 0.01 < *p* ≤ 0.05. **, *p* ≤ 0.01.

The antioxidant status and immune-inflammatory profiles in the serum and intestine are shown in Figure 5 and Figure 6. The concentrations of T-AOC, IL-22, and IgA in the serum of the piglets in the CGA group were significantly higher than those in the CON group (*p* < 0.05), while the concentrations of IL-1β, IL-6, TNF-α, and MDA in the serum of the piglets in the CGA group were significantly lower than those in the CON group (*p* < 0.05).

In the jejunum, the concentrations of T-AOC, T-SOD, and sIgA in the CGA group were significantly higher than those in the CON group (*p* < 0.05), while the concentrations of IL-1β, IL-6, TNF-α, and MDA in the CGA group were significantly lower than those in the CON group (*p* < 0.05).

In the ileum, the concentrations of T-AOC and CAT in the CGA group were significantly higher than those in the CON group (*p* < 0.05), while the concentrations of IL-1β and IL-6 in the CGA group were significantly lower than those in the CON group (*p* < 0.05).

### 2.7. Activation of the TLR4/NF-κB and Nrf2 Signaling Pathways

**Figure 7:**
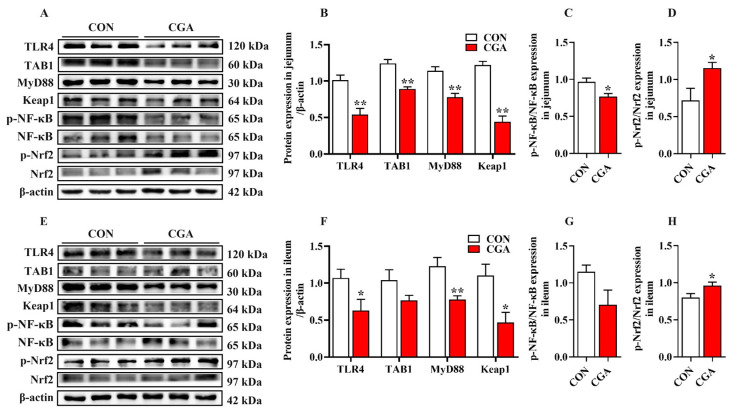
Effects of dietary CGA supplementation on the activation of the NF-κB and Nrf2 signaling pathways in the jejunum and ileum of piglets. (**A**–**D**) Jejunum; (**E**–**H**) ileum. *, 0.01 < *p* ≤ 0.05. **, *p* ≤ 0.01.

The relative protein abundance of the NF-κB and Nrf2 signaling pathways in the jejunum and ileum are shown in Figure 7. In the jejunum, the relative protein abundance of TLR4, TAB1, MyD88, and Keap1 in the CGA group was significantly lower than that in the CON group (*p* < 0.01). Meanwhile, the phosphorylation of NF-κB in the CGA group was significantly lower than that in the CON group (*p* < 0.05), while the Nrf2 phosphorylation in the CGA group was significantly higher than that in the CON group (*p* < 0.05). In the ileum, the relative protein abundance of TLR4, MyD88, and Keap1 in the CGA group was significantly lower than that in the CON group (*p* < 0.05). Moreover, the Nrf2 phosphorylation in the CGA group was significantly higher than that in the CON group (*p* < 0.05).

### 2.8. Correlation Analysis between Gut Microbiota, Oxidative Stress Indicators, and Immune-Inflammatory Factors

**Figure 8:**
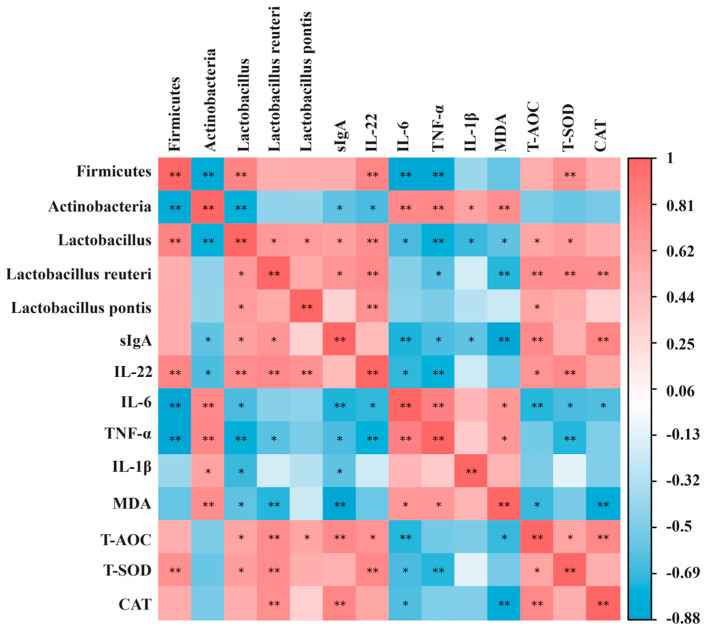
Correlation analysis between microorganisms, oxidative stress indicators, immunoglobulins, and inflammatory factors in the intestines of piglets. *, 0.01 < *p* ≤ 0.05. **, *p* ≤ 0.01.

The results of the Spearman correlation between the gut microbiota, oxidative stress indicators, and immune-inflammatory factors in the ileum of the piglets are shown in Figure 8. For the immune-inflammatory factors, the IL-22 secretion was significantly positively correlated with the abundance of Firmicutes (*p* < 0.01), and significantly negatively correlated with the abundance of Actinobacteria (*p* < 0.05). Regarding the Firmicutes, the IL-22 secretion was significantly positively correlated with the abundance of Lactobacillus at the genus level (*p* < 0.01), and *Lactobacillus reuteri* and *Lactobacillus pontis* at the species level (*p* < 0.01). The secretion of IL-6 and TNF-α was significantly negatively correlated with the abundance of Firmicutes (*p* < 0.01), while the secretion of IL-1β, IL-6, and TNF-α was significantly positively correlated with the abundance of Actinobacteria (*p* < 0.05). Regarding the Firmicutes, the secretion of IL-1β, IL-6, and TNF-α was significantly negatively correlated with the abundance of Lactobacillus at the genus level (*p* < 0.05). Moreover, the sIgA secretion was significantly positively correlated with the abundance of Lactobacillus at the genus level (*p* < 0.05) and *Lactobacillus reuteri* at the species level (*p* < 0.01), and was significantly negatively correlated with the abundance of Actinobacteria (*p* < 0.05). For the oxidative stress indicators, the T-SOD concentration was significantly positively correlated with the abundance of Firmicutes. Regarding the Firmicutes, the concentration of T-AOC and T-SOD was significantly positively correlated with the abundance of Lactobacillus at the genus level (*p* < 0.01) and *Lactobacillus reuteri* at the species level (*p* < 0.01). The MDA concentration was significantly positively correlated with the abundance of Actinobacteria (*p* < 0.01), and was negatively correlated with the abundance of Lactobacillus at the genus level (*p* < 0.05) and *Lactobacillus reuteri* at the species level (*p* < 0.01).

### 2.9. Network Pharmacological Analysis between CGA, Oxidative Stress, and Inflammation

**Figure 9:**
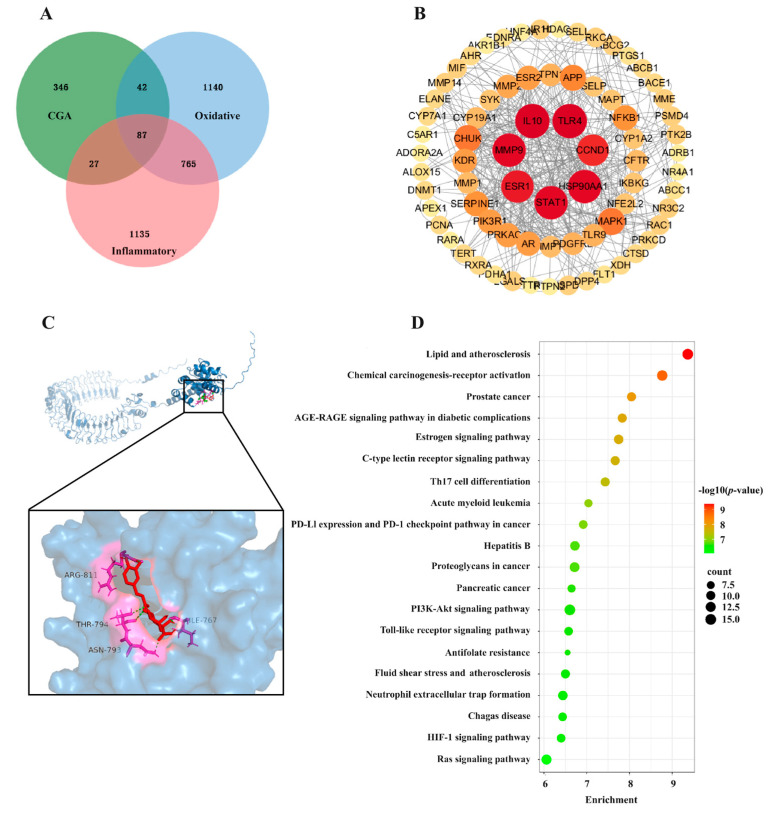
Network pharmacological analysis between CGA, oxidative stress, and inflammation. (**A**,**B**) Intersection analysis between CGA targets and the disease targets of oxidative stress and inflammation, as well as screening of core targets; (**C**) the schematic diagram of the interaction between CGA and TLR4; (**D**) the top 20 KEGG pathways.

As shown in Figure 9A, a total of 87 potential targets of CGA for alleviating oxidative stress and inflammation were obtained. The information of these 87 targets was entered into the String database, and the “Organisms” option was set to “Sus scrofa” to obtain the protein interaction network. Meanwhile, the Cytoscape 3.10 software was used to construct a PPI network based on this information. After a topological analysis of the network, the core target screening criterion was set to a Degree threshold of >34. A total of seven core targets were obtained (Figure 9B), including HSP90AA1, STAT1, ESR1, MMP9, TLR4, IL10, and CCND1. In order to elucidate the interaction between CGA and the target protein molecules, molecular docking simulations were conducted using the HOME for Researchers online platform (Figure 9C). The results showed that the free energy of CGA binding to TLR4 was −3.59 kcal/mol, which indicated that CGA could bind tightly to TLR4. In addition, the functions of 87 potential targets were predicted based on a GO and KEGG enrichment analysis (Figure 9D), and information on the top 20 signaling pathways was obtained. The results indicated that the “PI3K-Akt signaling pathway” and “Toll-like receptor signaling pathway” may be important pathways for CGA to regulate oxidative stress and inflammation in piglets.

## 3. Discussion

After weaning, factors such as dietary changes and immature development of the digestive tract and endocrine systems result in severe oxidative stress in the intestine of piglets [4,6,21]. Oxidative stress results in diarrhea, reduced feed intake, and growth retardation in weaned piglets [22,23]. CGA is a polyphenolic compound with potent antioxidant activity [11]. Accumulating evidence shows that CGA plays an important role in improving the growth performance, intestinal health, and disease resistance of piglets [10]. An early study showed that a sow diet supplemented with 300 mg/kg CGA during the lactation period increased the birth weight of neonatal pigs [20]. Chen et al. found that dietary supplementation with 1000 mg/kg CGA reduced the diarrhea incidence and improved the growth performance of piglets in a fourteen-day experiment [14]. They found that the enhancement of the intestinal antioxidant capacity and the improvement in digestion and absorption function may be key factors for CGA to improve the growth performance and reduce the diarrhea incidence of piglets [14]. In addition, the study showed that the growth performance of piglets fed diets supplemented with 250 or 500 mg/kg CGA was not markedly influenced [14]. Similarly, the present study found that dietary supplementation with 200 mg/kg CGA had no significant effect on the feed intake and final weight of piglets. However, this study found that dietary CGA supplementation significantly reduced the diarrhea incidence in piglets and showed a tendency to increase ADG. The different experimental results may be related to the different sources or dosages of CGA, as well as the feeding time.

Weaning is usually accompanied by intestinal barrier dysfunction in piglets, characterized by increased intestinal permeability [2]. The intestinal barrier of pigs can be divided into mechanical barriers (e.g., intestinal epithelial cells and tight junctions), immune barriers (e.g., mucins and defense proteins), and biological barriers (e.g., gut microbes) based on their structural and functional characteristics. The intestinal epithelial barrier is composed of intestinal epithelial cells and intercellular tight junctions [2]. Maintaining the integrity of the intestinal epithelial barrier is crucial for the digestion and absorption of nutrients, as well as resistance to pathogen invasion. The formation of tight junctions requires the involvement of several unique proteins, including the Claudin family, Occludin, and intracellular junction proteins (ZO-1), which, together with the intestinal epithelial cells, form a selective permeable barrier. However, weaning stress may induce the apoptosis of intestinal epithelial cells and inhibit the expression of tight junction proteins, ultimately increasing the permeability of the intestinal epithelial barrier [15,16]. A previous study showed that a diet supplemented with 500 mg/kg CGA improved the villus height and the ratio of the villus height to crypt depth in the intestines of piglets [24]. Consistently, this study found that CGA has a beneficial effect on improving the villous morphology of piglet intestines. Additionally, this study found that CGA upregulated the protein abundance of Claudin-1, Occludin, and ZO-1 in the intestine of piglets, which is consistent with the results of Chen et al. [14]. In addition, the immunohistochemical analysis showed that the protein abundance of Occludin and ZO-1 in the intestine of the piglets fed a diet supplemented with CGA was higher and located in the top region of the villi in the jejunum and ileum. Therefore, these results suggested that CGA could improve the intestinal epithelial barrier by maintaining the expression and localization of tight junction proteins, which may be partially responsible for the improved intestinal health associated with CGA in weaning-stressed piglets.

In addition to affecting the permeability of the intestinal epithelial barrier, CGA also regulates the secretion of porcine beta defensins [25]. Beta defensins are produced by Paneth cells, neutrophils, and epithelial cells in the gastrointestinal tract [26]. In this study, it was observed that CGA can increase the mRNA expression of beta defensins in the intestinal mucosa. This result indicates that CGA is an effective regulator of defense peptide genes. Notably, this observation contrasts with the absence of similar findings in prior CGA-related studies.

Gut microbes are an important component of the intestinal barrier in piglets [27]. Multiple studies have shown that weaning stress is one of the key factors affecting the composition and abundance of the gut microbiota in piglets [7]. The severe disturbance of the gut microbiota induces intestinal inflammation, damages the intestinal barrier, and then increases the diarrhea incidence in piglets [28]. In order to investigate the effect of CGA on bacterial colonization in the intestine of piglets, this study analyzed the microbiota in the piglet ileal contents through high-throughput 16S rRNA sequencing. The β-diversity analysis results showed that the microbiota in the CGA group tended to clearly separate from the CON groups, suggesting that CGA altered the microbial composition in the ileum of the piglets. Meanwhile, the results of the species composition analysis showed that CGA significantly increased the relative abundance of the bacteria from the phyla Firmicutes in the ileum of the piglets, which was in line with the previous results showing that dietary CGA supplementation significantly increased the relative abundance of Firmicutes in the cecal contents of piglets [29]. In this study, the changes in the gut microbiota of the piglets caused by CGA were mainly reflected in Lactobacillus, including *Lactobacillus reuteri* and *Lactobacillus pontis*. Similarly, previous studies have found a significant increase in the relative abundance of Lactobacillus in the colon or cecum of pigs fed CGA diets [24,29]. Compelling evidence suggests that *Lactobacillus reuteri* has excellent probiotic properties, including the positive modulation of piglet redox status and immune function [30,31], and competitive exclusion against pathogens [32]. Consistently, the results of the Spearman correlation between the gut microbiota, oxidative stress indicators, and immune-inflammatory factors in this study also showed that IL-22 and sIgA secretion was positively correlated with the abundance of *Lactobacillus reuteri*. Meanwhile, this study found that the T-AOC and T-SOD concentrations in the intestine were positively correlated with the abundance of *Lactobacillus reuteri*, while the MDA concentration was negatively correlated with the abundance of *Lactobacillus reuteri*. These results once again emphasize the beneficial role of *Lactobacillus reuteri* in improving intestinal inflammation and oxidative stress, which is consistent with previous reports [30,31]. Furthermore, *Lactobacillus reuteri* was found to be associated with the secretion of pBD2, pBD3, pBD114, and pBD129 in the intestine [33]. At present, there are few research reports on *Lactobacillus pontis*. In summary, dietary CGA supplementation enriched the abundance of beneficial bacteria in the intestines of piglets, which may have a positive effect on improving the intestinal barrier function of piglets.

As is well known, the intestinal barrier dysfunction in piglets during weaning is closely related to inflammation and oxidative stress [6]. Weaning stress can damage the intestinal health of piglets by upregulating the levels of reactive oxygen species (ROS) and pro-inflammatory cytokines in intestinal cells, and inhibiting the activity of antioxidant enzymes. Previous studies have reported that the excessive production of pro-inflammatory cytokines, especially IL-1β, IL-6, and TNF-α, directly disrupts the intestinal epithelial barrier [2]. Therefore, suppressing the excessive release of pro-inflammatory cytokines in the intestine is an effective strategy to alleviate intestinal diseases caused by weaning stress. This study found that CGA reduced the concentrations of IL-1β, IL-6, and TNF-α in the serum and intestine of piglets, and increased the IL-22 concentration, which is consistent with previous results [15]. Chen et al. reported that dietary supplementation with 1000 mg/kg CGA significantly reduced the mRNA expression of IL-1β, IL-6, and TNF-α in the small intestine of weaned piglets [15]. Furthermore, a previous study reported that CGA bolsters the intestinal barrier integrity in weaned rats by reducing the levels of pro-inflammatory factors in the intestine [34]. Collectively, these findings imply that dietary CGA supplementation may improve the intestinal health of piglets by inhibiting the over-release of pro-inflammatory cytokines and upregulating the levels of anti-inflammatory factors.

Oxidative stress is another key factor leading to intestinal barrier dysfunction. Under physiological conditions, multiple enzymes protect organisms from oxidative damage by maintaining a balance between the oxidative and antioxidant defense systems. It is widely recognized that SOD and CAT play a crucial role in the prevention of oxidative damage. Specifically, SOD is primarily responsible for scavenging superoxide radicals within cells, whereas CAT plays a key role in scavenging organic hydroxyl radicals. Previous studies have shown that CGA prevents H_2_O_2_-induced ROS production and provides more powerful antioxidant capacity than many other phenols by scavenging superoxide radicals. Palócz et al. [35] reported that CGA mitigated ROS accumulation in LPS-challenged IPEC-J2 cells. In vivo, dietary CGA supplementation increased the activity of GSH-Px and CAT in the intestine, which indicated the antioxidant capacity of CGA in weaned pigs [14]. In the present study, T-SOD and CAT, important components of the enzymatic antioxidant defense system, were significantly increased in piglets fed the CGA-supplemented diet, suggesting that CGA mitigated the excessive accumulation of reactive oxygen species. In addition, MDA, a secondary product of lipid oxidation, is closely related to cellular damage and has been widely recognized as an indicator to monitor the degree of lipid peroxidation [36]. In the present study, we found reduced MDA levels in the small intestine and serum of piglets supplemented with CGA diets, further indicating the antioxidant capacity of CGA in weaned pigs. Therefore, CGA appears to confer protection against oxidative stress in weaned pigs by bolstering antioxidant enzyme activities.

In order to further investigate the molecular mechanisms by which CGA reduces intestinal inflammation and oxidative stress, this study explored the impact of CGA on the activation of the TLR4/NF-κB and Nrf2 signaling pathways. As is well known, the TLR4/NF-κB signaling pathway is a classic pathway for regulating the cellular inflammatory response [37,38]. TLR4 is a representative member of the TLR family and is widely expressed in all types of intestinal cells [39]. Activated TLR4 can stimulate the activation of the NF-κB signaling pathway and upregulate the expression of inflammatory cytokine genes, including IL-1β, IL-6, and TNF-α [40,41]. Aligning with this, the network pharmacological analysis highlighted the “Toll-like receptor signaling pathway” as a pivotal route through which CGA modulates oxidative stress and inflammation in piglets. This study found that CGA reduced the abundance of TLR4, TAB1, MyD88, and NF-κB phosphorylation in the jejunum and ileum of the piglets, indicating that CGA weakened the activation of the TLR4/NF-κB signaling pathway. Previous studies have found that CGA can inhibit the synthesis of pro-inflammatory cytokines in human monocytes challenged with LPS and weaken the activation of TLR4 in LPS-challenged mice [40,41]. Dietary supplementation with CGA reduced the secretion of pro-inflammatory factors in the small intestine of weaned piglets, while inhibiting the mRNA expression of TLR4 and its downstream signal activation [15]. Based on these results, we speculate that CGA may partially alleviate intestinal inflammation by inhibiting the activation of the TLR4/NF-κB signaling pathway, reducing the secretion of intracellular inflammatory factors.

NF-E2-related factor 2 (Nrf2) and its endogenous inhibitor Kelch-like ECH-associated protein 1 (Keap1), as a ubiquitous intracellular defense mechanism, play a crucial role in regulating endogenous antioxidant enzyme levels [42]. Nrf2 is sequestered by cytoplasmic Keap1 under physiological conditions [43]. In the case of oxidative stress, Nrf2 is phosphorylated and detached from Keap1, followed by translocation to the nucleus, where it activates the transcription of antioxidant genes [44]. Growing evidence suggests that polyphenols possess the potential to activate the Nrf2/Keap1 signaling pathway and then upregulate the expression of antioxidative and cytoprotective genes in the intestine [45,46]. This study found that CGA can enhance the activity of antioxidant enzymes (T-SOD and CAT) in the jejunum and ileum of piglets by reducing the abundance of Keap1 and activating the Nrf2 signaling pathway. Consistently, previous studies have shown that dietary supplementation with CGA increased the mRNA expression of Nrf2 in the small intestine of weaned pigs, which was consistent with the increased activities of antioxidant enzymes [15]. Moreover, CGA has been reported to alleviate oxidative stress by increasing HO-1 levels [47]. Heme oxygenase 1 (HO-1) is an important antioxidant enzyme regulated by Nrf2 and plays a crucial role in regulating intracellular ROS levels [47]. HO-1 can reduce the expression of pro-inflammatory cytokines by inhibiting the activation of the NF-κB pathway [48]. However, the mechanism of crosstalk between the NF-κB signaling pathway and the Nrf2 signaling pathway in the intestine of weaned pigs needs further investigation.

## 4. Materials and Methods

### 4.1. Ethical Statement

All animal protocols used in this study were approved by the Animal Care and Use Committee of the Guangdong Academy of Agricultural Sciences (No. GAASIAS-2016-017, Guangzhou, China) and under the guidance of the Animal Management Regulations of China.

### 4.2. Animals, Diets, and Experimental Design

On the day of weaning, twenty-four piglets aged 21 days (6.80 ± 0.45 kg, Duroc × Landrace × Yorkshire) were randomly allotted to two groups based on initial body weight and gender, with six pens per group and each pen containing one male and one female. Piglets in the two groups were fed with a basal diet or a basal diet supplemented with 200 mg/kg CGA (containing ≥ 98%, Zhucheng Haotian Pharmaceutical Co., Ltd., Shandong, China), respectively. The usage of CGA was based on previous reports and a lower dose was selected [14]. Moreover, CGA was added to the diet as a component of the premix. The basal diets were formulated according to the nutrient requirements of 7 to 11 kg pigs recommended by the National Research Council (2012) [49], and the ingredients and nutrient levels are shown in Table 2. In addition, the experimental diet did not contain zinc oxide and antibiotics. All piglets were housed in a temperature-controlled nursery house, which was equipped with multiple pens. Each pen was laid with fully slatted floors (1.8 × 2.5 m), and equipped with a one-sided feeder and two stainless steel nipple drinkers. The room temperature for the first week was set at 28 ± 1 °C, with a daily decrease of 0.5 °C starting from the second week until 25 °C. The duration of the experiment was 21 days, during which all piglets had ad libitum access to feed and water.

### 4.3. Data and Sample Collection

The body weight of piglets was individually measured after 12 h of fasting on the morning of day 1 and day 22 of the experiment, and feed intake per pen was collected daily in order to calculate average daily feed intake (ADFI), average daily gain (ADG), and gain/feed ratio (G/F). The diarrhea rate was statistically analyzed based on the methods of Chen et al. [50]. Prior to weighing on day 22, blood samples were obtained from the anterior vena cava of fasting piglets (12 h) via vacuum tubes (GuangzhouKingMed Diagnostics Group Co., Ltd., Guangzhou, China), allowed to clot for 30 min, then centrifuged at 3000 r/min for 15 min to separate the serum. These serum samples were stored in a refrigerator at −80 °C until analysis. All piglets were euthanized with pentobarbital sodium (10 mg/kg) and slaughtered after the trial. Then, the duodenum, jejunum, and ileum were disconnected based on anatomical structure characteristics. The samples of duodenum, jejunum, and ileum for morphological analysis were fixed with 4% paraformaldehyde solution. After washing with 0.9% NaCl, the mucosal samples from the midpoint of these segments were gently scraped using glass slides, rapidly frozen in liquid nitrogen for gene expression analysis, and preserved at −80 °C. Moreover, ileal contents were harvested from each piglet and also stored at −80 °C.

### 4.4. Intestinal Morphology Analysis

The fixed duodenum, jejunum, and ileum tissues were retrieved from the paraformaldehyde solution. Then, the intestinal samples were embedded according to the introduction of standard paraffin embedding technology, and sectioned with a microtome (Leica RM2235, Wetzlar, Germany). Next, the sections were stained with hematoxylin and eosin for histopathological examination. Finally, the sections were scanned with a digital microscope scanner (Pannoramic 250, 3D HISTECH, Budapest, Hungary) to capture morphological features and images. At least 5 microscopic fields of view were randomly selected for each sample, and the parameters of villus height (V) and crypt depth (C) were measured by using the Image-pro Plus 6.0 Program, and the V/C ratio was calculated.

### 4.5. Chemical Analyses

Before analysis, the intestinal mucosa was physically fragmented. In short, 100 mg of intestinal mucosa was homogenized in 0.9 mL of 1 × PBS using a low-temperature homogenizer (Jingxin, Shanghai, China). Then, the homogenate was crushed in ultrasound for 5 min and centrifuged at 3000 r/min for 10 min at 4 °C to obtain the supernatant for further analysis. According to the instructions, the total superoxide dismutase (T-SOD), total antioxidant capacity (T-AOC), catalase (CAT), and malondialdehyde (MDA) in the serum and supernatant were detected using kits purchased from Nanjing Jiancheng Biological Research Institute (Table S1). The concentrations of interleukin (IL)-1β, IL-6, tumor necrosis factor (TNF)-α, IL-22, immunoglobulin (Ig) A, IgG, and secretory IgA (sIgA) in the serum and intestinal mucosa were determined with the Porcine ELISA Kits purchased from a commercial company (Jiangsu Meimian Industrial Co., Ltd., Yancheng, China). The information on kits used in this study is shown in Table S1.

### 4.6. Real-Time Quantitative PCR

Total RNA in the jejunal mucosa was extracted using the Trizol reagent (Invitrogen, Carlsbad, CA, USA) according to the manufacturer’s instructions. The quantity and quality of the RNA were analyzed by a NanoDrop ND-1000 spectrophotometer (NanoDrop Technologies, Wilmington, DE, USA). Then, the first-strand cDNA was synthesized using a PrimeScript 1st Strand cDNA Synthesis Kit (Takara, Tokyo, Japan). Next, these cDNAs were quantitatively analyzed using a Bio-Rad quantitative PCR instrument (C1000 Touch, Bio-Rad Laboratories, Richmond, CA, USA) with a SYBR Green PCR Master Mix (Bio-Rad, Laboratories, Richmond, CA, USA). All primers were designed using the Primer 5.0 software (Premier Bio-soft International, San Francisco, CA, USA) and synthesized by Sangon Biotech Co., Ltd. (Shanghai, China). The primer sequences are shown in Table 3. Samples were normalized to β-actin, and the relative mRNA expression was analyzed by the 2^−ΔΔCt^ method.

### 4.7. Western Blot Analysis

Total protein from the intestinal mucosa was extracted using a mixture of RIPA lysis buffer containing 1% PMSF protease inhibitor (Biosharp Life sciences, Anhui, China) and 1% phosphatase inhibitors. Then, the BCA Protein Assay Kit (Beyotime, Shanghai, China) was used to analyze the concentration of total protein. Next, an amount of total protein was mixed with protein loading buffer according to the instructions and denatured at 100 °C to make a sample for electrophoresis. In order to separate proteins of different molecular sizes in the samples, the samples containing 25 μg of total protein were loaded onto 10% SDS-PAGE gels (Beyotime, Shanghai, China) and subjected to electrophoresis. After electrophoresis, proteins of different molecular sizes were electrotransferred to a 4.5 cm × 8.5 cm polyvinylidene difluoride membrane (Millipore, Bedford, MA, USA) and blocked with 5% skimmed milk for 2 h. After washing with buffer, these membranes were sequentially incubated in primary and secondary antibody dilutions to allow the proteins to be labeled with antibodies. The antibody information is shown in Table S2. Finally, the band signals on these membranes were detected using ChemiDoc XRS imaging equipment (Thermo Fisher Scientific, Wilmington, DE, USA) and an ECL chemiluminescence solution (Beyotime, Shanghai, China). The gray values of these bands were evaluated and quantified by Image J Software (ImagePro Plus 6.0, Media Cybernetics, Inc., Rockville, MD, USA). The relative protein abundance or the phosphorylation of the target protein was calculated.

### 4.8. Immunohistochemical

Intestinal tissue sections were subjected to the following protocol: Initial fixation in 10% neutral buffered formalin, followed by xylene incubation for dewaxing, a descending ethanol series for dehydration, and antigen retrieval in citrate buffer solution. Subsequently, immunostaining was conducted using a streptavidin-biotin-peroxidase complex (ABC) method. Detailed information regarding the primary and secondary antibodies utilized is presented in Table S2.

### 4.9. 16S rRNA Gene Sequencing of Ileal Microbiota

Microbial genomic DNA was isolated from ileal contents using the Cetyltrimethylammonium Bromide (CTAB) method, and DNA quality was analyzed using agarose gel electrophoresis. Subsequently, the V3-V4 region of the 16S rRNA gene was amplified using universal primers, including the forward primer 341F (5′-ACTCCTACGGGAGGCAGCA-3′) and reverse primer 806R (5′-TCGGACTACHVGGGTWTCTAAT-3′). Sequencing analyses of 16S rRNA were based on amplicon sequence variants (ASVs) and were conducted on the Thermo Fisher Ion SSTM XL platform (Novogene, Beijing, China). Low-quality bases from all raw reads were trimmed off using Cutadapt software (version 1.9.1), and the reads were then assigned to individual samples based on barcode sequences. Then, operational taxonomic unit (OTU) clustering was performed using Uparse software (version 7.0.1001), with OTUs defined at a 97% sequence identity threshold using USEARCH software version v.10. The alpha diversity was analyzed using Shannon’s index, Simpson index, and Chao l index. The beta diversity was analyzed using principal component analysis (PCoA) and principal coordinate analysis on unweighted UniFrac distance matrices.

### 4.10. Network Pharmacological Analysis

Possible targets for CGA were obtained based on the Traditional Chinese Medicine Systems Pharmacology Database and Analysis Platform (TCMSP). The following databases were used to obtain potential targets for CGA: Comparative Toxicogenomics Database (https://ctdbase.org/ (accessed on 6 May 2024)), GeneCards (https://www.genecards.org/ (accessed on 10 May 2024)), OMIM (https://omim.org/ (accessed on 13 May 2024)), Therapeutic Target Database (https://db.idrblab.net/ttd/ (accessed on 9 May 2024)), and DisGeNET (https://www.disgenet.org/ (accessed on 16 May 2024)). Meanwhile, the gene information related to inflammation and oxidative stress was obtained from these databases. Then, a Venn diagram was created based on bioinformatics technology, and the crossover genes were copied into Excel as potential targets [51]. Next, the target information for mitigating oxidative stress and inflammation with CGA was imported into the string database (https://string-db.org/ (accessed on 22 May 2024)) to procure Protein–Protein Interactions (PPIs) and TSV files [52]. The TSV files were opened with Cytoscape 3.10 software (Case Viewer 2.3, 3D HISTECH, Budapest, Hungary) to visualize protein interaction networks, followed by topological assessments to pinpoint central targets. Kyoto Encyclopedia of Genes and Genomes (KEGG) pathway enrichment and Gene ontology (GO) analysis of the intersection targets was performed based on bioinformatics techniques. Finally, the HOME for Researchers online platform was used to build the molecular docking diagram of CGA and TLR4.

### 4.11. Statistical Analysis

An individual pen served as one experimental unit. Data were analyzed using GraphPad Prism version 8.0 (GraphPad Software, San Diego, CA, USA) and significance was evaluated using a T-test. Values are presented as mean ± SEM. 0.01 < *p* ≤ 0.05 indicates statistically significant differences (*), *p* ≤ 0.01 indicates a statistically very significant difference (**), and 0.05 < *p* ≤ 0.1 indicates a trend of significant change.

## 5. Conclusions

This study found that CGA can reduce the secretion of pro-inflammatory factors and increase the activity of antioxidant enzymes by inhibiting the TLR4/NF-κB signaling pathway and activating the Nrf2/Keap1 signaling pathway, ultimately alleviating intestinal inflammation and oxidative stress and promoting intestinal health in piglets. Additionally, this study found that dietary supplementation with CGA alleviated intestinal inflammation and oxidative stress by increasing the abundance of *Lactobacillus reuteri* and *Lactobacillus pontis* in the piglet intestines. These findings enrich the theoretical basis for CGA to improve weaned intestinal health and are of great significance for guiding the development of nutritional strategies for weaned piglets.

## Figures and Tables

**Table 1:** Effects of CGA on the growth performance of weaned piglets (*n* = 6).

Item	CON	CGA	SEM	*p*-Value
Initial BW, kg	6.85	6.73	0.19	0.836
Final BW, kg	13.21	13.96	0.46	0.131
ADG, g/d	302.70	372.00	12.17	0.096
ADFI, g/d	454.82	497.11	15.86	0.164
G/F	0.67	0.75	0.01	0.567
Diarrhea rate, %	29.17	17.78	2.78	0.016

CON, control group; CGA, basal diet supplemented with 200 mg/kg CGA; BW, body weight; ADG, average daily gain; ADFI, average daily feed intake; G/F, gain/feed ratio.

**Table 2:** Ingredient composition and nutrient levels of the basal diet.

Ingredients	%	Nutrient Levels ^2^	
Corn	41.21	NE, MJ/kg	10.88
Enzyme-treated soybean meal	19.46	CP, %	22.73
Expanded soybean	11.00	SID CP, %	18.43
Low-protein whey powder	10.00	SID Lys, %	1.43
Whey protein concentrate	4.00	SID Met + Cys, %	0.78
Fishmeal	3.00	SID Thr, %	0.84
Soybean oil	2.82	SID Trp, %	0.26
Sucrose	2.00	SID Ile, %	0.85
Soybean hulls	2.00	SID Val, %	0.91
Lysine HCl	0.33	SID Leu, %	1.64
DL-Methionine	0.14	SID Lys/ME, g/MJ	5.49
L-Threonine	0.09	Ca, %	0.79
Calcium hydrophosphate	0.80	STTD P, %	0.62
NaCl	0.35	Na, %	0.31
Limestone powder	1.80		
Premix ^1^	1.00		
Total	100.00		

^1^ Supplied per kilogram of complete diet: Fe, 120 mg; Cu, 10 mg; Zn, 120 mg; Mn, 35 mg; I, 0.25 mg, Se, 0.2 mg; vitamin A, 8000 IU; vitamin D3, 1000 IU; vitamin E, 30 mg; vitamin K3, 2 mg; vitamin B1, 2 mg; vitamin B2, 6 mg; vitamin B6, 4.0 mg; vitamin B12, 0.02 mg; niacin, 25 mg; calcium pantothenate, 10 mg; folic acid, 1.0 mg; biotin, 0.25 mg. ^2^ Nutrient levels are calculated according to the NRC (2012) [49].

**Table 3:** Primer sequences used in this study.

Genes	Prime Sequence (5′-3′)	GenBank	Product Size (bp)
*MUC*	F: CTGCTCCGGGTCCTGTGGGA	XM_021082584.1	101
	R: CCCGCTGGCTGGTGCGATAC		
*pBD-1*	F: ACCGCCTCCTCCTTGTATTC	NM_213838.1	150
	R: CACAGGTGCCGATCTGTTTC		
*pBD-2*	F: CCAGAGGTCCGACCACTACA	NM_214442.2	88
	R: GGTCCCTTCAATCCTGTTGAA		
*PG1*	F: GTAGGTTCTGCGTCTGTGTCG	NM_001123149.2	166
	R: CAAATCCTTCACCGTCTACCA		
*β-actin*	F: TGCGGGACATCAAGGAGAAGC	XM_021086047	273
	R: ACAGCACCGTGTTGGCGTAGAG		

Abbreviations in italics indicate gene names. *MUC*, mucin; *pBD*, porcine beta defensins; *PG1*, Protegrin-1.

## Data Availability

Please contact the author if further information is required.

## Supplementary Materials

The following supporting information can be downloaded at: https://www.mdpi.com/article/10.3390/ijms25189954/s1.
