# Supplementary material for: Chlorogenic Acid Enhances the Intestinal Health of Weaned Piglets by Inhibiting the TLR4/NF-κB Pathway and Activating the Nrf2 Pathway
Source: Int J Mol Sci. 2024 Sep 15;25(18):9954. doi: 10.3390/ijms25189954 (PMC11432128; doi:10.3390/ijms25189954)
Supplement: Supplementary file 1 [file ijms-25-09954-s001.zip › ijms-3180316-supplementary.pdf]

Supplementary Material

Table S1 Elisa kit information in the study

Items	Company	Product code
T-AOC	Nanjing Jiancheng, China	A015-2-1
T-SOD	Nanjing Jiancheng, China	A001-3-2
CAT	Nanjing Jiancheng, China	A007-1-1
MDA	Nanjing Jiancheng, China	A003-1-2
IL-1β	Jiangsu Meimian, China	MM-042201
IL-6	Jiangsu Meimian, China	MM-041801
TNF-α	Jiangsu Meimian, China	MM-038301
IL-22	Jiangsu Meimian, China	MM-123101
sIgA	Jiangsu Meimian, China	MM-3623401

Table S2 Antibody information used in the study

Items	Company	Product code
TLR4	Proteintech, USA	66350-1
TAB1	Cell Signaling Technology, USA	3226S
MyD88	Cell Signaling Technology, USA	4283S
Nrf2	LifeSpan, USA	A003-1-2
p-Nrf2	Cell Signaling Technology, USA	MM-0422O1
Keap1	Cell Signaling Technology, USA	48768T
NFκB	proteintech, USA	10745-1-AP
p-NFκB	Cell Signaling Technology, USA	3033S
ZO-1	Cell Signaling Technology, USA	13663S
Occludin	Abcam, USA	ab31721
Claudin-1	Abcam, USA	ab15098
β-Actin	Beyotime, Shanghai, China	AF5003
